# A Simulation of Low and High Cycle Fatigue Failure Effects for Metal Matrix Composites Based on Innovative *J*_2_-Flow Elastoplasticity Model

**DOI:** 10.3390/ma10101126

**Published:** 2017-09-24

**Authors:** Zhaoling Wang, Heng Xiao

**Affiliations:** 1 School of Mathematics and Information Sciences, Weifang University, Weifang 261061, China; 2 School of Mechanics and Construction Engineering, MOE Key Lab of Disaster Forecast and Control in Engineering, Jinan University, Guangzhou 510632, China

**Keywords:** metal matrix composites, high and low cycle fatigue, finite deformations, new elastoplastic equations, unified failure criterion, direct simulation

## Abstract

New elastoplastic $J_{2}$-flow constitutive equations at finite deformations are proposed for the purpose of simulating the fatigue failure behavior for metal matrix composites. A new, direct approach is established in a two-fold sense of unification. Namely, both low and high cycle fatigue failure effects of metal matrix composites may be simultaneously simulated for various cases of the weight percentage of reinforcing particles. Novel results are presented in four respects. First, both the yield condition and the loading–unloading conditions in a usual sense need not be involved but may be automatically incorporated into inherent features of the proposed constitutive equations; second, low-to-high cycle fatigue failure effects may be directly represented by a simple condition for asymptotic loss of the material strength, without involving any additional damage-like variables; third, both high and low cycle fatigue failure effects need not be separately treated but may be automatically derived as model predictions with a unified criterion for critical failure states, without assuming any ad hoc failure criteria; and, finally, explicit expressions for each incorporated model parameter changing with the weight percentage of reinforcing particles may be obtainable directly from appropriate test data. Numerical examples are presented for medium-to-high cycle fatigue failure effects and for complicated duplex effects from low to high cycle fatigue failure effects. Simulation results are in good agreement with experimental data.

## 1. Introduction

With improved fatigue and impact performance [1,2,3], metal matrix composites (MMCs) have in the recent past been used toward reducing weights of structural components and parts. For instance, Al-matrix composites with ceramic TiC reinforcing particles exhibit excellent mechanical strength with improved fatigue resistance and creep resistance [4,5,6,7] and have been used in automobile industry. A detailed review for literature of fatigue of reinforced composites and recent applications may be found in LLorca [8].

The fatigue behavior of MMCs is known to be mainly responsible for fracture and failure of structural components and parts made of such materials. Toward effectively assessing structural reliability and safety under complex service conditions, rational and realistic modeling of the fatigue failure behavior for MMCs is accordingly essential and has attracted intensive interest in both theoretical and experimental studies.

In the past decades, numerous investigations into fracture and failure of MMCs have been carried out from various standpoints. Details may be found in the survey articles [9,10,11,12]. Certain representative results for MMCs are as follows. Quast et al. [13] investigated the out-of-phase thermomechanical fatigue behavior of Ultra SCS-6/Ti-24Al-17Nb-xMo (at.%) MMCs; the fatigue behavior of two 2009/SiC/15p-T4 DRA (discontinuously reinforced aluminum) composites in the very high cycle fatigue was investigated by Huang et al. [14]; Ni et al. [15] studied residual stresses and high cycle fatigue properties of friction stir welded SiCp/AA2009 composites; fatigue failure mechanisms of microsphere Al2O3-Al particulate MMCs were determined from examination of the fracture surfaces and the crack profiles by Park et al. [16]; Sivananth et al. [7] evaluated the load bearing behavior of titanium carbide reinforced aluminum matrix composites and their suitability for automotive application; Feng et al. [17] determined the relationship between the applied stress and fatigue life of a SiC fiber-reinforced titanium matrix composite and examined the fracture surfaces to study the fatigue damage and fracture failure mechanisms using SEM; and the creep–fatigue behavior of aluminum alloy-based MMC has been modeled using an idealized plane strain model [18]. Moreover, many other related studies may be found in [19,20,21,22,23,24,25].

According to usual approaches, however, issues in a few respects have been left outstanding. First, augmented constitutive structures with additional damage-like variables should be assumed from different standpoints [9,10,11,12] and, second, various ad hoc criteria for fracture and failure, etc. should be introduced on an empirical basis [9,10,11,12]. Third, because of disparate failure features under low and high cycle conditions, such as the complicated duplex effect, etc., as will be explained in Section 2, the low and high cycle fatigue failure effects have to be treated, separately, and it does not appear that a unified simulation of both effects is currently available. In particular, fatigue failure effects of MMCs should be simulated individually for various cases of the weight percentage of reinforcing particles.

In a most recent study [26,27], it has been demonstrated that a new, direct approach may be proposed toward modeling fatigue, fracture and failure effects of metals, etc. Toward this objective, the central idea is to establish new elastoplasticity models into which the fatigue failure behavior is incorporated as inherent constitutive features. With such new models, it has been shown [26,27] that the usual notion of yielding becomes irrelevant with a gradual, smooth transition from the elastic to the plastic state in a more realistic sense. As a result, the fatigue failure behavior may be automatically derived as a direct consequence of certain simple asymptotic properties of the strength quantities incorporated.

In this article, new elastoplastic $J_{2}$-flow constitutive equations at finite deformations in the general framework of the most recent study above will be proposed for the purpose of simulating the fatigue failure behavior for MMCs. A new, direct approach will be established in a two-fold sense of unification. Namely, both low and high cycle fatigue failure behaviors of MMCs may be simultaneously simulated for various cases of the weight percentage of reinforcing particles. Novel results will be presented in four respects. First, both the yield condition and the loading–unloading conditions in a usual sense need not be involved but may be automatically incorporated into inherent features of the proposed constitutive equations; second, low-to-high cycle fatigue behaviors may be directly represented by a simple condition for asymptotic loss of the material strength, without involving any additional damage-like variables; third, both high and low cycle fatigue failure effects need not be separately treated but may be automatically derived as model predictions with a unified criterion for critical failure states, without assuming any ad hoc failure criteria; and, finally, explicit expressions for each incorporated model parameter changing with the weight percentage of reinforcing particles may be obtainable directly from appropriate test data. Numerical examples will be presented for medium-to-high cycle fatigue effects and for complicated duplex effects from low to high cycle fatigue failure effects and will be compared with test data.

The main content of this contribution is arranged as follows. In Section 2, certain main concepts on the fatigue failure for MMCs are introduced for the purpose of motivating the succeeding development and, in Section 3, a new elastoplastic $J_{2}$-flow model will be proposed in the aforementioned novel sense; thermodynamic consistency of this new model will be demonstrated in explicit, identical sense in Section 4; in Section 5, a failure criterion in unified form will be derived from the proposed model; predictions of the proposed model under uniaxial cyclic loadings up to failure will be then studied and numerical examples will be presented and compared with test data in Section 6; and, finally, some remarks will be given in Section 7.

## 2. Main Concepts on the Fatigue Failure of MMCs

In this section, main concepts of *S*-*N* curves for the fatigue failure of MMCs under cyclic loading conditions will be introduced for the purpose of motivating and explaining the subsequent development.

### 2.1. Typical S-N Curves with Fatigue Strength

**Figure 1:**
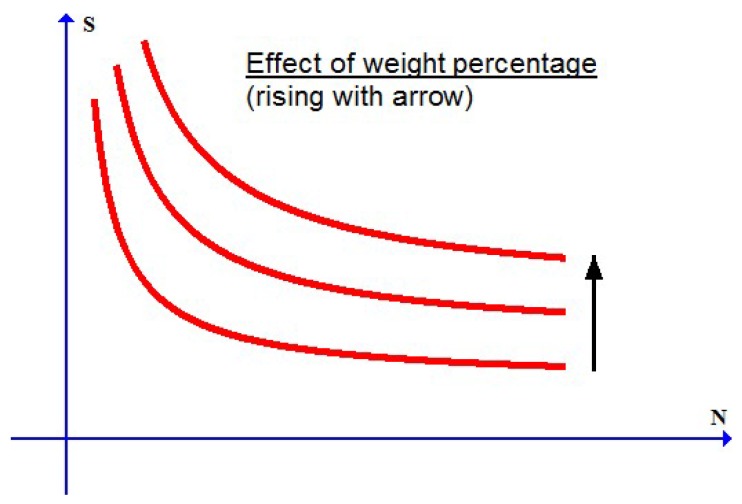
*S*-*N* curves for MMCs (metal matrix composites) with different weight percentages of reinforcing particles (S: stress amplitude; N: number to failure).

Typical *S*-*N* curves for the fatigue failure of MMCs under cyclic loading conditions are schematically shown in Figure 1 for different cases of the weight percentage of reinforcing particles. As reported in experiments (cf., e.g., [4,5,6,7]), the *S*-*N* curve goes up with increasing weight percentage of reinforcing particles. Each curve becomes nearly flat at very high cycles. This implies that, whenever the stress amplitude is below a limiting value, referred to as the fatigue limit, the cycle number to failure will become indefinitely large and the fatigue failure in this case actually will not take place.

### 2.2. Duplex Feature from Low to High Cycle Fatigue Failure

**Figure 2:**
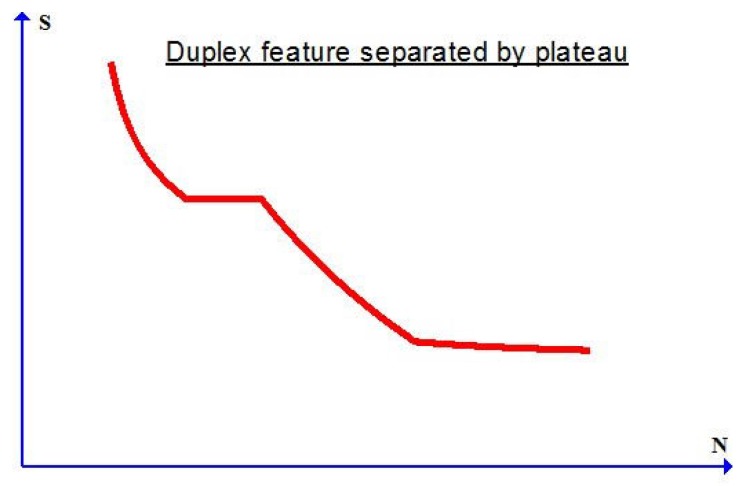
S-N curve displaying the duplex feature from low to high cycle fatigue failure.

Arising from distinct crack nucleation modes indicated later on, the *S*-*N* curve may exhibit a complex feature known as the duplex feature. As schematically shown in Figure 2, there appears a plateau part separating the two parts for low-to-medium cycle fatigue failure and for medium-to-high cycle fatigue failure in the *S*-*N* curve.

It is found (cf., e.g., [25]) that the two parts in the *S*-*N* curve with the duplex feature are related to the surface-induced crack nucleation mode (low to medium cycle fatigue failure) and the subsurface-inclusion-induced crack nucleation mode (medium to high cycle fatigue failure) separately. Details for this complex duplex effect may be found in [25].

As in Figure 1, the *S*-*N* curve in Figure 2 with the foregoing duplex feature will be different for various values of the weight percentage of reinforcing particles.

### 2.3. The Main Objective for Model Simulation

It is required that a realistic constitutive model is established to simulate the fatigue failure effects of **MMCs** in a sense of achieving agreement with experimental data. It appears that, for various cases of the weight percentage of reinforcing particles, a unified simulation of the foregoing features poses a challenging issue. In particular, that may be the case for the fatigue failure behavior with the duplex feature. As mentioned earlier, the low cycle fatigue effect and the high cycle fatigue effect have to be treated, separately.

In the sequel, a new elastoplastic $J_{2}$-flow model will be proposed and a unified simulation will be established based on this new model.

## 3. Innovative Elastoplastic $J_{2}$-Flow Model for MMCs

Consider an elastoplastic body undergoing finite deformations. Let ***F*** and ***L*** be the deformation gradient and the velocity gradient and, moreover, let ø, ***W*** and ***D*** be the Kirchhoff stress, the vorticity tensor and the stretching tensor, namely,

$$ø=Jo\;e,\;J=\det F\;L=\dot{F}\cdot F^{-1},$$

$$W=\frac{1}{2}\left(L-L^{T}\right),\;D=\frac{1}{2}\left(L+L^{T}\right),$$

where oe and *J* are the Cauchy stress (true stress) and the volumetric ratio, respectively.

In the past decades, various formulations of finite elastoplastic deformations have been developed (cf., e.g., [28]). In what follows, we direct attention to the self-consistent objective Eulerian rate-type formulation [29,30,31,32,33] based on the following additive separation of the strechting ***D***:

(1) $$D=D^{e}+D^{p},$$

with the elastic part $D^{e}$ and the plastic part $D^{p}$. These two parts should be formulated by two Eulerian objective rate equations and will be given in two steps below, separately.

### 3.1. Elastic Rate Equation

A Eulerian elastic rate equation is given at the first step in the following self-consistent sense: prior to the initial yielding with $D^{e}=D$, it is exactly integrable to really deliver a finite hyperelastic relation. For MMCs with small elastic strain, by extending the rate form of the well-known Hooke’s law, a self-consistent elastic rate formulation may be given below [29,30,31,32,33]:

(2) $$D^{e}=\frac{1}{2G}\left({\overset{˚}{ø}}^{\log}-\frac{\upsilon }{1+\upsilon }\left(\mathrm{tr}\dot{ø}\right)I\right).$$

In the above, υ and *G* are the Poisson ratio and the shear modulus and, moreover, ${\overset{˚}{ø}}^{\log}$ is the co-rotational logarithmic rate of the Kirchhoff stress ø below:

(3) $${\overset{˚}{ø}}^{\log}=\dot{ø}+ø\cdot \Omega^{\log}-\Omega^{\log}\cdot ø,$$

with the logarithmic spin

(4) $$\Omega^{\log}=W+\sum_{r\neq s=1}^{n}\left(\frac{1+\frac{b_{r}}{b_{s}}}{1-\frac{b_{r}}{b_{s}}}+\frac{2}{\ln\frac{b_{r}}{b_{s}}}\right)B_{r}\cdot D\cdot B_{s},$$

where $b_{1}$, ⋯, $b_{n}$ and $B_{1}$ , ⋯, $B_{n}$ are the *n* distinct eigenvalues of the left Cauchy–Green tensor $B=F\cdot F^{T}$ and the corresponding eigenprojections of ***B***, respectively. Details may be found in [34].

### 3.2. New Flow Rule

A flow rule for the plastic part $D^{p}$ is given at the next step. To this end, the normality flow rule of the following form is used (cf., e.g., [35,36]):

(5) $$D^{p}=\rho \frac{\overset{˘}{f}}{\overset{˘}{h}}\frac{\partial f}{\partial ø},$$

where the plastic indicator ρ, the yield function *f*, and the loading factor $\xi =\overset{˘}{f}/\overset{˘}{h}$ are explained and given below.

According to the classical theory of elastoplasticity, the plastic indicator ρ in Equation (5) is assumed to be given by the idealized approximation below: no plastic deformation would be induced prior to yielding, whereas plastic deformation would be induced only in the case when yielding is attained and maintained. Specifically, ρ=0 for the unloading case and ρ=1 for the loading case (refer to, e.g., [35] for details).

As explained in [26,37], each usual elastoplasticity model with the plastic indicator ρ prescribed above could not simulate fatigue failure effects under cyclic loading conditions. This may particularly be the case for the so-called high cycle fatigue failure with the stress amplitude far below the initial yield limit. Instead of assuming the above plastic indicator ρ in a sense of idealized approximation, a new plastic indicator ρ may be introduced in a more realistic sense below. Namely, the plastic deformation may be induced at any stress level with a continuously changing plastic indicator 0≤ρ≤1, which becomes close to 0 for the stress point staying far away from the yield surface f=0 and close to 1 for the stress point staying in the vicinity of the yield surface f=0.

With the above idea in mind, we may introduce a more realistic plastic indicator ρ as follows:

(6) $$\begin{array}{c} \left\{\begin{array}{c} \rho =\;\frac{1.5J_{2}}{q^{2}}e^{-m\left(1-\frac{1.5J_{2}}{q^{2}}\right)},\;\\ m=m_{1}+\left(m_{2}-m_{1}\right){\left[\cosh\gamma_{0}\left(\frac{1.5J_{2}}{s_{0}^{2}}-1\right)\right]}^{-1},\;\\ J_{2}=\mathrm{tr}{\tilde{ø}}^{2},\; \end{array}\right. \end{array}$$

where $m_{1}>0$, $m_{2}>0$, $\gamma_{0}>0$ and $s_{0}>0$ are positive material parameters, the $\tilde{ø}$ is the deviatoric part of ø, and the *q* is known as the stress limit and will be given in Equation (9) later on. The meanings of these parameters will be explained slightly later.

With the new plastic indicator ρ given in Equation (6), a new flow rule may be given as follows [26,37,38,39]:

(7) $$D^{p}=\rho \frac{\xi +|\xi |}{2}\frac{\partial f}{\partial ø}.\;$$

In the above, the *f* is the von Mises function of the form:

(8) $$\left\{\begin{array}{cc} f & =g-r,\\ g & =\frac{1}{2}J_{2},\\ r & =\frac{1}{3}q^{2},\; \end{array}\right.$$

with the stress limit of the form below [39]:

(9) $$q=\frac{1}{2}q_{0}\left[1-\tanh\beta \left(\frac{\kappa }{\kappa_{c}}-1\right)\right].$$

In the above, $q_{0}$, β, and $\kappa_{c}$ are positive material parameters and the κ is the plastic work prescribed by

(10) $$\dot{\kappa }=ø:D^{p}.\;$$

In addition, the loading factor ξ in the flow rule Equation (7) is given by

(11) $$\xi =\frac{\overset{˘}{f}}{\overset{˘}{h}},$$

with

(12) $$\overset{˘}{f}=2G\tilde{ø}:D,$$

(13) $$\overset{˘}{h}=\frac{2}{3}J_{2}\left(3G+qq^{\prime }\right),\;q^{\prime }=\frac{\partial q}{\partial \kappa }.$$

### 3.3. New $J_{2}$-Flow Model

The new $J_{2}$-flow model is obtained by combining Equations (1) and (2) and Equations (7)–(13), as given below:

(14) $$\frac{{\overset{˚}{ø}}^{\log}}{2G}=\frac{\nu }{1-2\nu }\left(\mathrm{tr}D\right)I+D-\frac{1.5G\rho }{3G+qq^{\prime }}J_{2}^{-1}(\tilde{ø}:D+|\tilde{ø}:D\left|\right)\tilde{ø},\;$$

(15) $$\dot{\kappa }=\frac{1.5G\rho }{3G+qq^{\prime }}(\tilde{ø}:D+|\tilde{ø}:D\left|\right),\;$$

where the plastic indicator ρ and the stress limit *q* are given by Equations (6) and (9), respectively.

The new elastoplastic $J_{2}$-flow model proposed above is fully free in a sense without involving the yield condition as well as the loading–unloading conditions. The new model gives rise to plastic flow at any non-zero stress level whenever $\tilde{ø}:D>0$. Whenever the stress reaches such a level that the yield limit in classical sense is met, plastic strain will become dominant. Otherwise, it may be negligibly small for a stress level within the yield limit. Consequences implied by the new model may be found in [26,27,37].

As explained in [26,27,37], fatigue failure effects may be automatically incorporated as inherent constitutive features of the new model. Here, the essential point lies in the fact that, from a phenomenological standpoint, the physical essence of material failure would be just loss of the stress-bearing capacity attendant with fully developed plastic flow. The conditions expressing this fact have been presented in [26] in a general constitutive framework. The plastic indicator ρ and the stress limit *q* given in Equations (6) and (9) are just particular forms meeting these general conditions.

In the above, the plastic factor ρ ensures accumulation of the plastic work at any stress level and, accordingly, it plays an essential role in characterizing the fatigue failure under both low and high cycle conditions. As such, the new elastoplastic model established can automatically simulate fatigue failure behavior without any additional variables and related equations and conditions. In fact, a unified criterion for critical failure states will be derived as a direct consequence, as will be shown in Section 5. Detail in this respect may be found in [26,27] and in Section 5.

It should be pointed out that the stress limit *q* given in Equation (9) is taken from the previous study [37], whereas the plastic indicator ρ given in Equation (6) is new and includes the following form:

(16) $$\rho =\frac{1.5J_{2}}{q^{2}}e^{-m_{1}\left(1-1.5J_{2}/q^{2}\right)}$$

as a particular case with $m_{2}=m_{1}$. The above form has been used (cf., [39]) in simulating certain aspects of the fatigue failure for metals. The new plastic indicator as given in Equation (6) is essential for a unified simulation of low-to-high cycle fatigue failure effects with the duplex feature indicated in Figure 2.

**Figure 3:**
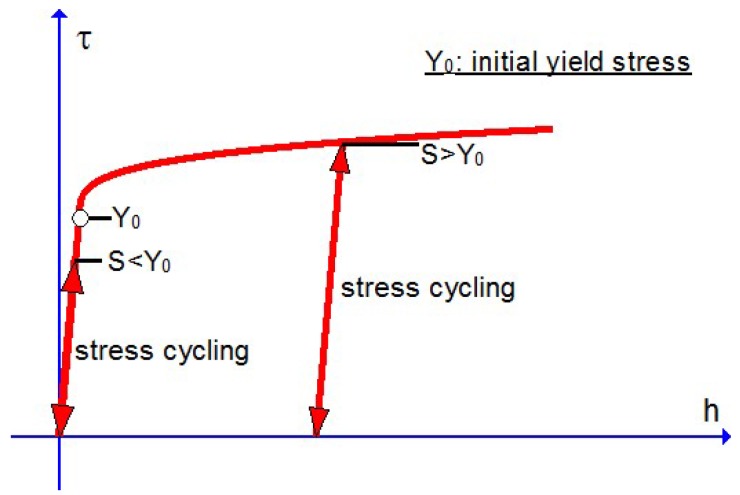
Responses of the usual model with the classical plastic indicator under the uniaxial stress cycling from 0 to *S* and then back to 0 keep the same for both $S<Y_{0}$ and $S>Y_{0}$.

**Figure 4:**
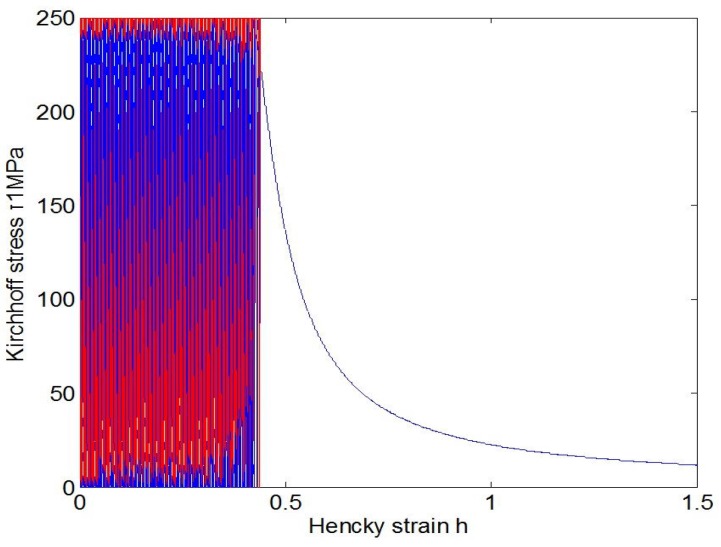
Ratcheting response of the new model with the plastic indicator Equation (16) under uniaxial stress cycling from 0 to 250 MPa and then back to 0, in which a critical failure state is reached at the number of 4032 cycles.

**Figure 5:**
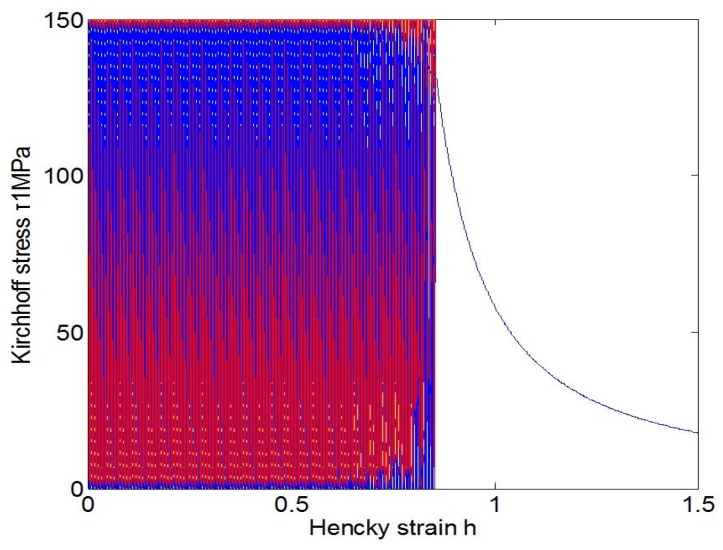
Ratcheting response of the new model with the plastic indicator Equation (16) under uniaxial stress cycling from 0 to 150 MPa and then back to 0, in which a critical failure state is reached at the number of 40,746 cycles.

As indicated in the last subsection, fatigue effects under cyclic loadings could not be simulated by the usual $J_{2}$-flow model with the classical plastic indicator taking values 0 or 1, but may be automatically simulated by the new $J_{2}$-flow model with a new plastic indicator. Toward highlighting this difference, the responses of the usual and the new model under the uniaxial stress cycling from 0 to *S* and then back to 0 are shown in Figure 3, Figure 4 and Figure 5, separately. Under the stress cycling at issue, the usual model with the classical plastic indicator could predict no fatigue effects, let alone the eventual failure, whereas the new model may automatically predict the ratcheting effect up to the eventual failure, with no assumed failure criteria associated with any additional damage-like variables.

### 3.4. Physical Meanings of the Material Parameters

**Figure 6:**
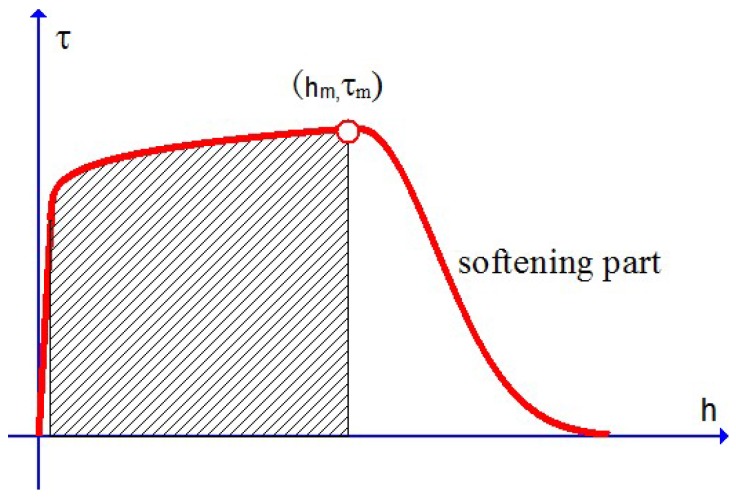
Uniaxial tensile curve explaining the three parameters in Equation (9): the tensile strength $\tau_{m}$ and the area of the shading part provide approximate values of the maximum stress limit $q_{0}$ and the critical plastic work $\kappa_{c}$, respectively, and the slope of the softening part specifies the softening index β.

The main feature of the stress limit *q* given by Equation (9) is as follows: for a fairly large β, the stress limit *q* actually yield a constant value, $q_{0}$, before the plastic work κ reaches a critical value slightly greater than $\kappa_{c}$. After the plastic work κ reaches the critical value $\kappa_{c}$, the stress limit goes rapidly down to vanish with softening effect up to eventual failure. The dimensionless parameter β is referred to as the softening index of the following property: the greater the β is, the more rapidly the stress limit *q* goes down to vanish. All these three parameters may be evaluated with a uniaxial tensile curve with softening effect, as illustrated in Figure 6.

The parameter m>0 in the plastic indicator Equation (6) is referred to as the plastic index, which specifies the magnitude of plastic strain induced at each stress level. The parameters $m_{1}$ and $m_{2}$ in Equation (6) control the magnitude of plastic strain as the stress amplitude is above or below $s_{0}$. On the other side, the parameter $s_{0}$ represents stress level at which the pleteau part in Figure 2 locates and is called the plateau stress. In addition, the parameter $\gamma_{0}$ controls the slope of the plateau part and is referred to as the transition index.

In what follows, it will be demonstrated that the fatigue failure behavior of MMCs with the main features indicated in the last section may be in a unified manner simulated by the new $J_{2}$-flow model by finding out suitable values of the above material parameters and the elastic constants E=2G(1+ν) and ν. Toward this objective, it should be noted that each parameter introduced will rely on the weight percentage of reinforcing particles. Let this weight percentage be designated by 0≤ω≤1. Then,

(17) $$\begin{array}{c} \left\{\begin{array}{c} E=E(\omega ),\;\nu =\nu (\omega ),\;\\ m_{1}=m_{1}\left(\omega \right),\;m_{2}=m_{2}\left(\omega \right),\;s_{0}=s_{0}\left(\omega \right),\;\gamma_{0}=\gamma_{0}\left(\omega \right),\;\\ q_{0}=q_{0}\left(\omega \right),\;\beta =\beta \left(\omega \right),\;\kappa_{c}=\kappa_{c}\left(\omega \right).\; \end{array}\right. \end{array}$$

As will be shown in Section 6, the above parameters changing with the weight percentage ω may be determined from suitable test data by means of direct procedures.

## 4. Thermodynamic Consistency of the New Model

Since fatigue failure effects are always associated with strong dissipation, it may be essential that a constitutive model simulating such effects should be placed on the rigorous thermodynamic ground, in order to guarantee the physical reality and reasonableness. On account of this, in this section, we are going to demonstrate that the new model proposed in the last section identically meets the universal restrictions imposed by the thermodynamic laws. To this end, following the main procedures in [33,38], we demonstrate that the specific entropy function and the free energy function, here denoted η and Ψ, may be presented in explicit forms, so that the second law with non-negative intrinsic dissipation, viz., the Clausius–Duhem inequality below [33,38]:

(18) $$\tau :D-\dot{\Psi }-\eta \dot{T}-\frac{J}{T}q\cdot \nabla T\geq 0$$

is fulfilled for any given forms of the constitutive quantities incorporated in the proposed model. In the above, T>0 is the absolute temperature, q is the heat flux vector and *J* is the deformation Jacobian.

With a positive function φ=φ(κ,T) monotonically increasing with increasing plastic work κ, i.e.,

(19) $$\varphi \left(0,T\right)=0,\;\frac{\partial \varphi }{\partial \kappa }>0,\;$$

we construct the following forms of the specific entropy and the Helmholtz free energy:

(20) $$\eta =\frac{\partial^{2}\bar{W}}{\partial \tau \partial T}:\;\tau -\frac{\partial \Psi }{\partial T},\;$$

(21) $$\Psi =\psi \left(T\right)+\frac{\partial \bar{W}}{\partial \tau }:\tau -\bar{W}+\kappa -\varphi \left(\kappa ,T\right),\;$$

where the temperature-dependent quantity ψ(T) represents the specific heat capacity and the $\bar{W}$ is the complementary elastic strain-energy function of quadratic form below:

(22) $$\bar{W}=\frac{1}{4G}\mathrm{tr}\tau^{2}-\frac{\nu }{2E}{\left(\mathrm{tr}\tau \right)}^{2},$$

with Young’s modulus E=2G(1+ν) (here, both *E* and *G* are allowed to be temperature-dependent in a broad sense). Then, it follows from Equations (1), (2), (7), (10) and Equations (18)–(21) that the intrinsic dissipation

(23) $$\begin{array}{c} D=\tau :D-\dot{\Psi }-\eta \dot{T} \end{array}$$

is given by

(24) $$\begin{array}{c} D=\frac{1}{2}\rho J_{2}\left(\xi +|\xi |\right)\frac{\partial \varphi }{\partial \kappa }\geq 0.\; \end{array}$$

Thus, from the above and the fact that the heat flux q should be opposed to the temperature gradient, namely, -q·∇T>0, we deduce that the Clausius–Duhem inequality Equation (18) is identically fulfilled.

Thus, we come to the conclusion that the proposed model is thermodynamically consistent for any given forms of the plastic indicator ρ>0 and the stress limit *q*.

## 5. Unified Criterion for Critical Failure States

The main objective of this section is to derive a unified criterion for fatigue failure effects from the new model proposed. Toward the above objective, we reformulate the loading factor ξ (cf., Equation (11)) in terms of the strain rate (stretching) ***D***, as given by Equations (11)–(13), in an other form in terms of the stress rate ${\overset{˚}{ø}}^{\log}$. With ξ>0, this will be done as follows.

First, from Equations (7) and (8) and Equations (10)–(13) with ξ>0, we deduce

(25) $$\dot{\kappa }=\rho J_{2}\frac{\overset{˘}{f}}{\overset{˘}{h}}$$

and

(26) $$2G\rho J_{2}\frac{\overset{˘}{f}}{\overset{˘}{h}}=\overset{˘}{f}-\hat{f},$$

where the $\overset{˘}{f}$ and $\overset{˘}{h}$ are given by Equations (12) and (13) and, moreover, the $\hat{f}$ is given by

(27) $$\hat{f}=\tilde{\tau }:{\overset{˚}{ø}}^{\log}.$$

Then, we obtain

(28) $$J_{2}\frac{\overset{˘}{f}}{\overset{˘}{h}}=\frac{\hat{f}}{2G\left(1-\rho \right)+\frac{2}{3}qq^{\prime }}.$$

Now, it becomes clear that, for each non-vanishing stress rate, the plastic work κ may grow at an infinite rate whenever the denominator in Equation (28) becomes vanishing, namely,

(29) $$2G\left(1-\rho \right)+\frac{2}{3}qq^{\prime }=0.$$

As the plastic work invariably accumulates, the above condition will be satisfied and a critical failure state is reached. Immediately following such a state, the plastic work κ will exceed the critical value $\kappa_{c}$ and the stress limit *q* (cf., Equation (9)) will go rapidly down to vanish with further development of plastic flow. Then, eventual failure follows.

It may be worthwhile to note that the criterion Equation (29) is derived in a broad sense for every process of multi-axial deformations. With the above understanding, the usually known fatigue failure effect under cyclic loading conditions becomes evident. Indeed, according to the new model proposed, plastic strain will be induced at every non-vanishing stress level, albeit very small, and, accordingly, the plastic work κ will constantly grow as a loading cycle repeats itself. As such, the criterion Equation (29) will be met and a critical failure state in the foregoing will be reached, thus leading to eventual failure.

The above facts are evidenced in Figure 4 and Figure 5. Under the stress cycling in these figures, the critical failure states are reached after 4032 cycles for the stress amplitude 250 MPa and after 40,746 cycles for the stress amplitude 150 MPa, separately, and then the eventual failure follows in both cases of low and high cycle fatigue .

A direct, unified approach toward simulating fatigue failure effects may be established based on the proposed new model with the unified criterion Equation (29). In particular, low and high cycle fatigue failure effects may be simultaneously simulated, as will be shown in the numerical examples given in the next section.

## 6. Numerical Examples for Model Validation

In this section, numerical examples will be provided for simulating certain experimental data in literature. Results will be given for two cases. The one is concerned with medium-to-high cycle fatigue failure effects for aluminum-matrix composites with ceramic TiC reinforcing particles, while the other with low-to-high cycle fatigue failure effects for stainless steel displaying the complex duplex feature.

The test data at issue are for uniaxial cyclic loadings. In what follows, governing equations for the uniaxial loading case will be derived in Section 6.1. Then, simulation results will be presented and compared with test data in Section 6.2 and Section 6.3.

### 6.1. Governing Equations for the Uniaxial Case

Consider a cylindrical sample undergoing uniaxial deformations. Let τ be the axial Kirchhoff stress and let *l* and *a* be the stretch ratios in the lateral direction and the axial direction, respectively. Then, from Equations (14) and (15), we can derive the rate equations for the plastic work κ and the axial Hencky strain h=lna as follows:

(30) $$\dot{\kappa }=\frac{2}{3}\left(1+\nu \right)\frac{q_{0}^{2}}{E}\frac{[\bar{\tau }\dot{h}]\rho }{1+\frac{qq^{\prime }}{3G}-\left[\bar{\tau }\dot{h}\right]\rho }\;\bar{\tau }\dot{\bar{\tau }},$$

(31) $$\dot{h}=\frac{q_{0}}{E}\frac{1+\frac{qq^{\prime }}{3G}-\frac{1-2\nu }{3}\left[\bar{\tau }\dot{h}\right]\rho }{1+\frac{qq\prime }{3G}-\left[\bar{\tau }\dot{h}\right]\rho }\;\dot{\bar{\tau }},$$

where $\bar{\tau }=\tau /q_{0}$ and

(32) $$\rho =\frac{{\bar{\tau }}^{2}q_{0}^{2}}{q^{2}}\exp\left[-m\left(1-\frac{q_{0}^{2}}{q^{2}}{\bar{\tau }}^{2}\right)\right],\;$$

(33) $$m=m_{1}+\left(m_{2}-m_{1}\right){\left[\cosh\gamma_{0}\left(\frac{q_{0}^{2}}{s_{0}^{2}}{\bar{\tau }}^{2}-1\right)\right]}^{-1},\;$$

(34) $$qq^{\prime }=-\frac{1}{4}q_{0}^{2}\frac{\beta }{\kappa_{c}}\frac{1-\tanh\beta (\frac{\kappa }{\kappa_{c}}-1)}{\cosh^{2}\beta \left(\frac{\kappa }{\kappa_{c}}-1\right)},$$

$$\left[x\right]=\left\{\begin{array}{cc} 1, & \;\mathrm{for}\;x>0,\\ 0, & \;\mathrm{for}\;x\leq 0. \end{array}\right.$$

In deriving Equations (30) and (31), the relation below for the lateral stretch ratio *l* has been used:

(35) $$\ln l=-\frac{1}{2}h+\frac{1-2\nu }{2}\frac{q_{0}}{E}\bar{\tau }.$$

For any given process of the axial loading, namely, τ=τ(t), the response of the axial Hencky strain h=h(t) may be derived from Equations (30) and (31) with Equations (32)–(34). In particular, consider here a uniform deformation process consisting of stress cycles. Let *S* be any given stress amplitude. At each cycle, the stress grows up to *S* and then down to uS with a ratio u<1. A cycle with u=-1 is called a symmetric one. At each cycle, the plastic flow is induced in the two processes from 0 to *S* and from *S* to uS, separately. The fatigue life under the cyclic process at issue is the cycle number to failure, denoted *N*, for which a critical failure state is attained. It is determined by the criterion Equation (29).

Numerical results will be given below for two cases, separately.

### 6.2. Fatigue Failure for Al-Matrix Composites with TiC Reinforcing Particles

We first simulate the data in [7] for Al-matrix composites with TiC reinforcing particles. In this reference, stress cycles with the ratio u=0.1 indicated at the end of Section 6.1 is taken into consideration and medium-to-high cycle fatigue failure data are provided for four cases of the weight percentage of TiC reinforcing particles, namely, for ω=0, 0.1, 0.12, 0.15, separately.

Of the nine parameters listed in Equation (17), the two elastic constants *E* and ν are given in [7]. The values of the other parameters are found in fitting the data for the four cases separately. The values of the three dimensionless parameters ν (Poisson ratio), β (softening index) and $\gamma_{0}$ (transition index) are actually constant for the four cases and given by

$$\nu =0.3,\;\beta =5,\;\gamma_{0}=50.$$

The values of the other parameters change with the weight percentage ω and listed in Table 1.

**Figure 7:**
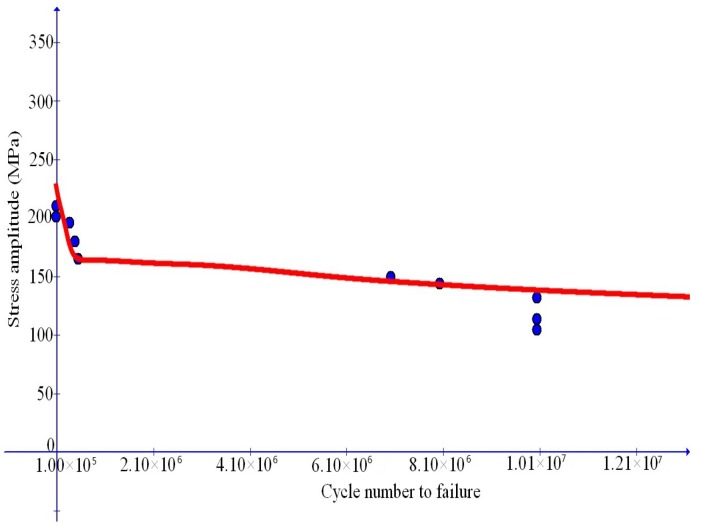
Comparison of simulation results with fatigue failure data (dots) from Sivananth et al. [7] for unreinforced LM6 alloy.

**Figure 8:**
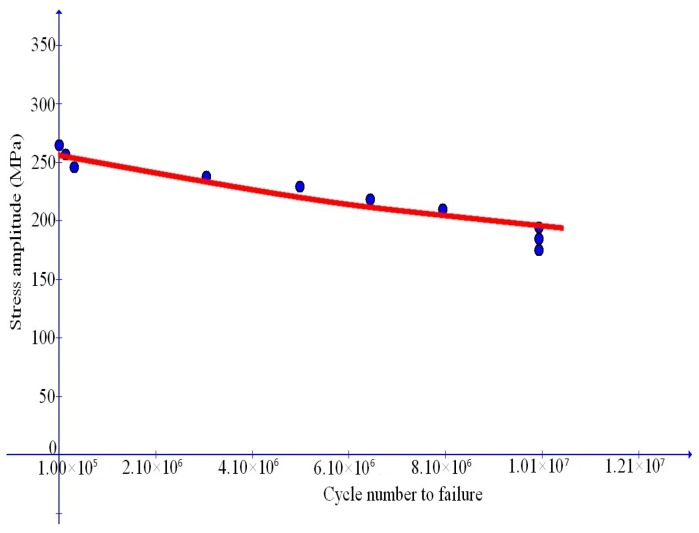
Comparison of simulation results with fatigue failure data (dots) from Sivananth et al. [7] for Al-matrix composite with 10% (wt) TiC reinforcing particles.

**Figure 9:**
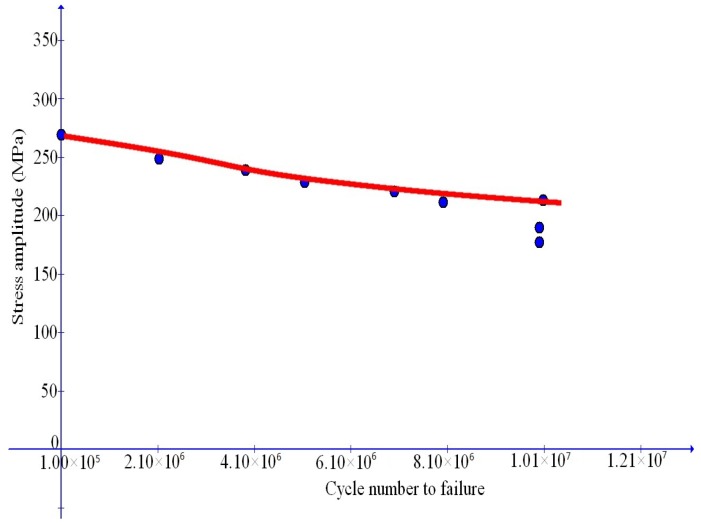
Comparison of simulation results with fatigue failure data (dots) from Sivananth et al. [7] for Al-matrix composite with 12% (wt) TiC reinforcing particles.

**Figure 10:**
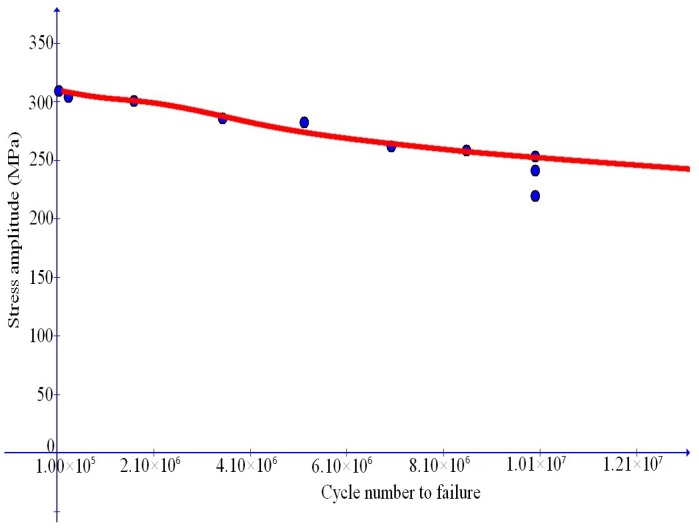
Comparison of simulation results with fatigue failure data (dots) from Sivananth et al. [7] for Al-matrix composite with 15% (wt) TiC reinforcing particles.

Simulation results are depicted in Figure 7, Figure 8, Figure 9 and Figure 10 and compare well with test data. The values of the Young’s modulus *E* are available from [7]. The maximal stress limit $q_{0}$ may be evaluated at the tensile strength in the uniaxial test, while the values of the plateau stress $s_{0}$ may be estimated from those slowly changing data. The $\kappa_{c}$ may be approximated by the stress work as the stress reaches the tensile strength in the uniaxial case. Finally, refined values of these parameters as well as the values of the dimensionless parameters $m_{1}$ and $m_{2}$ may be obtained in such a manner that test data should be closely fitted.

By means of direct procedures, a unified expression of each parameter as a function of the weight percentage ω (cf., Equation (17)) may be constructed, which reproduces the parameter values identified at $\omega =\omega_{1},\cdots ,\omega_{N}$. At first sight, this may be done by means of usual polynomial interpolating procedures. However, usual interpolating polynomials could not serve this purpose, since they are known to exhibit the unreasonable Runge phenomenon of oscillatory nature at the end points.

Toward bypassing the above issue, a new approach is suggested here to combine any given number of parameter values into a unified, reasonable expression. Instead of usual interpolating polynomials, now each parameter χ=χ(ω) changing with the weight percentage ω may be given as follows:

(36) $$\chi =\sum_{r=1}^{N-1}I_{r}\left(\omega \right)\varphi_{r}\left(\omega \right).\;$$

In the above, the $\varphi_{r}\left(\omega \right)$ are *N* smooth functions of localized nature and given by

(37) $$\varphi_{r}\left(\omega \right)=\frac{1}{2}\left[\tanh\left(3000(\omega -\omega_{r})+3\right)-\tanh\left(3000(\omega -\omega_{r+1})+3\right)\right],\;$$

and, besides, the $I_{r}\left(\omega \right)$ are the N-1 parabolic splines given by

(38) $$I_{r}\left(\omega \right)=\chi_{r}+g_{r}\left(\omega -\omega_{r}\right)+\xi_{r}\left(\omega -\omega_{r}\right)\left(\omega -\omega_{r+1}\right),\;$$

where $g_{1}$, ⋯, $g_{N-1}$ are given by

(39) $$\begin{array}{c} g_{r}=\frac{\chi_{r+1}-\chi_{r}}{\omega_{r+1}-\omega_{r}},\;r=1,\cdots ,N-1,\; \end{array}$$

and $\xi_{1}$, ⋯, $\xi_{N-1}$ are determined by the iterative procedures below:

(40) $$\begin{array}{c} \xi_{r}=\frac{g_{r}-g_{r-1}}{\omega_{r+1}-\omega_{r}}-\frac{\omega_{r}-\omega_{r-1}}{\omega_{r+1}-\omega_{r}}\;\xi_{r-1},\;r=2,\cdots ,N-1,\; \end{array}$$

with

(41) $$\xi_{1}=\frac{g_{2}-g_{1}}{\omega_{2}-\omega_{1}}.\;$$

It may be readily shown that the following continuity conditions for the derivative are satisfied at the node points $\omega =\omega_{2},\cdots ,\omega_{N-1}$:

$$I_{r}^{\prime }\left(\omega_{r+1}\right)=I_{r+1}^{\prime }\left(\omega_{r}\right),\;r=1,\cdots ,N-1.$$

In particular, Equations (41) and (40) with r=2 yield

(42) $$\xi_{2}=0.\;$$

Each function $\varphi_{r}$ above actually takes the constant value 1 within the interval $\left[\omega_{r},\omega_{r+1}\right)$ and goes rapidly down to vanish outside the just mentioned interval.

**Figure 11:**
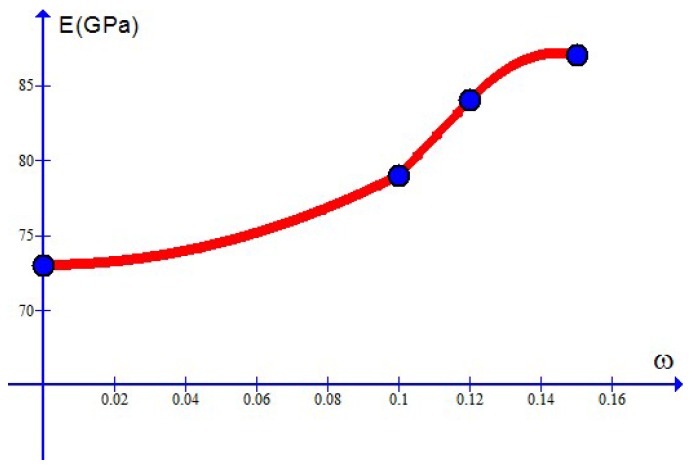
Young’s modulus *E* changing with the weight percentage ω (dots for simulation results).

**Figure 12:**
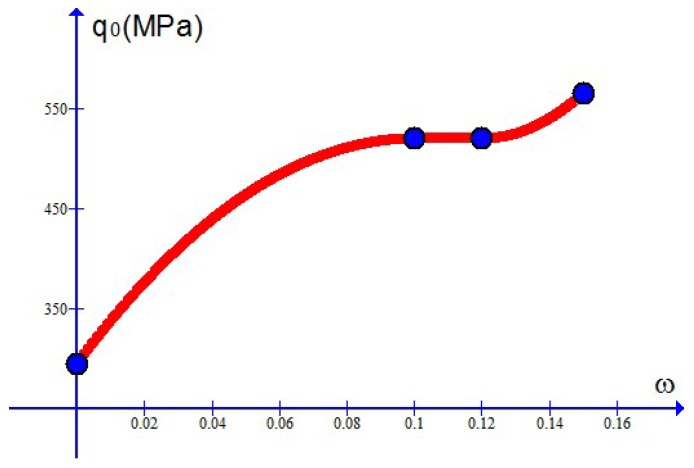
The maximum stress limit $q_{0}$ changing with the weight percentage ω (dots for simulation results).

**Figure 13:**
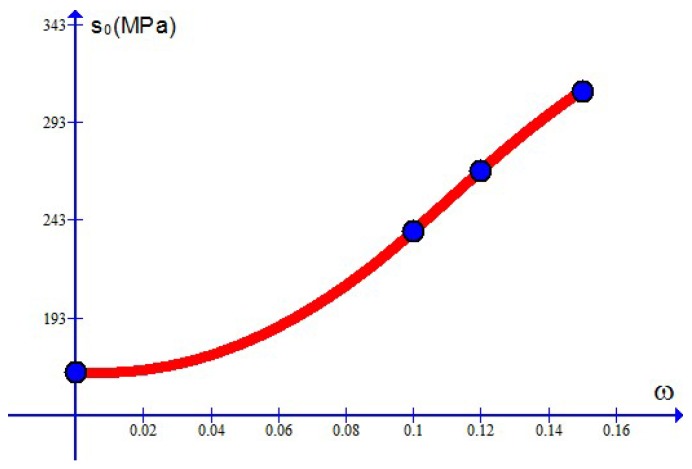
The plateau stress $s_{0}$ changing with the weight percentage ω (dots for simulation results).

For the case treated here, *N* is given by 4 and the parameter values $\chi_{r}=\chi \left(\omega_{r}\right)$ at $\omega_{1}=0$, $\omega_{2}=0.1$, $\omega_{3}=0.12$, $\omega_{4}=0.15$ are listed in Table 1 for each parameter χ. As examples, the curves of the Young’s modulus E=E(ω), the maximum stress limit $q_{0}=q_{0}\left(\omega \right)$ and the plateau stress $s_{0}=s_{0}\left(\omega \right)$ are depicted in Figure 11, Figure 12 and Figure 13, separately. The curve of the critical plastic work $\kappa_{c}=\kappa_{c}\left(\omega \right)$ is of the same shape as the curve $q_{0}=q_{0}\left(\omega \right)$ and not shown here.

### 6.3. Low-to-High Cycle Fatigue Failure with Duplex Feature

In this subsection, model prediction will further be validated by simulating low-to-high cycle fatigue failure data displaying the complex duplex effect as indicated in Figure 2. Toward this end, the most recent data in [25] is taken into consideration. In this reference, data are provided for JIS SUS630 stainless steel under symmetric stress cycles with the ratio u=-1.

As mentioned in Section 2, a unified simulation of the fatigue failure behavior with the complex duplex effect poses a challenging issue. In fact, it is found [25] that a duplex *S*-*N* curve consists of two parts resulting separately from the surface-induced crack nucleation mode and the subsurface-inclusion-induced crack nucleation mode. This implies that the just mentioned two micro-mechanisms of distinct nature need be treated toward modeling the duplex effect in a unified sense.

Now, the foregoing low-to-high cycle fatigue failure data are in a unified manner simulated with the new approach proposed. The parameter values are found by fitting the data at issue and given below:

$$E=191\;\mathrm{GPa},\;\nu =0.3,\;m_{1}=12.7,\;m_{2}=7,\;q_{0}=1056\;\mathrm{MPa},\;\kappa_{c}=300\;\mathrm{MPa},\;\beta =5/\mathrm{MPa},\;\gamma_{0}=50,\;s_{0}=750\;\mathrm{MPa}.$$

**Figure 14:**
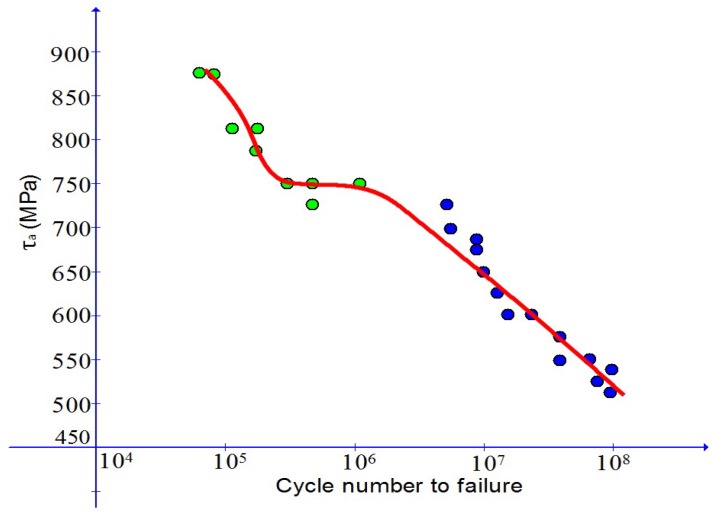
Comparison of simulation results with data (dots) from Mohd et al. [25] with duplex features from low to high cycle fatigue failure.

Comparison of the simulation results with test data is shown in Figure 14 and good agreement with data is achieved.

## 7. Conclusions

In the previous sections, new elastoplastic $J_{2}$-flow equations have been proposed for the purpose of achieving a direct, unified simulation of fatigue failure effects for MMCs. A unified criterion for critical failure states has been derived as model prediction, which is applicable to all cases of multiaxial fatigue failure. Numerical examples for model validation have been presented separately for medium-to-high cycle fatigue failure effects and for low-to-high cycle fatigue failure effects with complex duplex feature. It has been demonstrated that simulation results are in good agreement with experimental data.

As indicated in Section 4, the new approach proposed is applicable for simulating fatigue failure effects in a broad case of general multi-axial deformations. Here, simulation results are given for the uniaxial case and compared with test data under uniaxial cyclic loadings. Further study will be needed to compare model predictions with test data for fatigue failure effects under various types of multi-axial cyclic and non-cyclic loadings and to treat deformation modes with rotational effects, such as simple shear and torsion, etc. In particular, non-uniform deformations and stresses need be treated for fatigue failure effects displaying in realistic components made of MMCs. For such realistic problems, it is expected that finite element modeling should be used together with the new model proposed here.

Moreover, it may be of interest to simulate fatigue failure effects for fiber-reinforced composite materials and for polymeric solids. Initial anisotropies need be treated for the former, while appreciable rate-dependent effects should be considered for the latter. It is expected that, in conjunction with the previous work for initially anisotropic elastoplastic solids [33] and for rate-dependent elastoplastic models of soft solids [38], the approach proposed here may be extended to study these further aspects. Results will be reported elsewhere.

## Figures and Tables

**Table 1:** Parameter values changing with the weight percentage.

ω (%)	*E* (GPa)	$q_{0}$ (MPa)	$\kappa_{c}$ (MPa)	$s_{0}$ (MPa)	$m_{1}$	$m_{2}$
0	73	295	80	165	9.0	6.2
10	79	520	95	237	8.4	3.0
12	84	520	95	268	9.1	6.0
15	87	565	100	309	9.6	6.2
